# Mesothelial cells in tissue repair and fibrosis

**DOI:** 10.3389/fphar.2015.00113

**Published:** 2015-06-09

**Authors:** Steven E. Mutsaers, Kimberly Birnie, Sally Lansley, Sarah E. Herrick, Chuan-Bian Lim, Cecilia M. Prêle

**Affiliations:** ^1^Centre for Cell Therapy and Regenerative Medicine, School of Medicine and Pharmacology, University of Western Australia and Harry Perkins Institute of Medical Research, Nedlands, WA, Australia; ^2^Institute for Respiratory Health, Centre for Asthma, Allergy and Respiratory Research, School of Medicine and Pharmacology, University of Western Australia, Nedlands, WA, Australia; ^3^Institute of Inflammation and Repair, Faculty of Medical and Human Sciences and Manchester Academic Health Science Centre, University of Manchester, Manchester, UK

**Keywords:** inflammation, coagulation and fibrinolysis, tissue repair and fibrosis, extracellular matrix, mesothelial-to-mesenchymal transition, post-operative adhesion, idiopathic pulmonary fibrosis

## Abstract

Mesothelial cells are fundamental to the maintenance of serosal integrity and homeostasis and play a critical role in normal serosal repair following injury. However, when normal repair mechanisms breakdown, mesothelial cells take on a profibrotic role, secreting inflammatory, and profibrotic mediators, differentiating and migrating into the injured tissues where they contribute to fibrogenesis. The development of new molecular and cell tracking techniques has made it possible to examine the origin of fibrotic cells within damaged tissues and to elucidate the roles they play in inflammation and fibrosis. In addition to secreting proinflammatory mediators and contributing to both coagulation and fibrinolysis, mesothelial cells undergo mesothelial-to-mesenchymal transition, a process analogous to epithelial-to-mesenchymal transition, and become fibrogenic cells. Fibrogenic mesothelial cells have now been identified in tissues where they have not previously been thought to occur, such as within the parenchyma of the fibrotic lung. These findings show a direct role for mesothelial cells in fibrogenesis and open therapeutic strategies to prevent or reverse the fibrotic process.

## Introduction

Mesothelial cells form a monolayer, known as the mesothelium, that line the pleural, peritoneal, and pericardial cavities, with visceral and parietal surfaces covering the internal organs and body wall, respectively. They attach to a thin basement membrane supported by sub-serosal connective tissue, and are bathed in a small volume of serosal fluid that resembles an ultrafiltrate of plasma containing blood proteins, sugars, resident inflammatory cells, and various enzymes (Mutsaers, 2002).

Mesothelial cells synthesize and secrete lubricants including glycosaminoglycans and surfactant to prevent friction and adhesions forming between adjacent parietal and visceral surfaces. They play critical roles in the maintenance of serosal homeostasis in response to injury, inflammation, and immunoregulation (reviewed in Mutsaers and Wilkosz, 2007). Mesothelial cells are also central cells in serosal repair, secreting inflammatory mediators, chemokines, growth factors, and extracellular matrix (ECM) components.

Mesothelial cells display different phenotypes which, depending on their location and state of activation, are likely to reflect functional differences. Although morphologically they resemble epithelial cells and possess many epithelial characteristics; surface microvilli, apical/basal polarity, cytokeratins, and junctional complexes, embryologically they derive from the mesoderm and express mesenchymal features including vimentin and desmin (Batra and Antony, 2014). Upon stimulation they can also undergo morphological and functional changes consistent with an epithelial-to-mesenchymal transition (EMT; Yanez-Mo et al., 2003; Aroeira et al., 2007; Perez-Lozano et al., 2013) which has recently been termed mesothelial-to-mesenchymal transition (MMT; Sandoval et al., 2010).

The ability of mesothelial cells to undergo MMT suggests that the mesothelium is a likely source of fibrogenic cells during serosal inflammation and tissue repair and therefore play important roles in pleural and peritoneal fibrosis and adhesion formation. In addition, it has been hypothesized that mesothelial cells may be a source of (myo)fibroblasts in interstitial lung fibrosis (Decologne et al., 2007; Zolak et al., 2013; Karki et al., 2014; Chen et al., 2015).

**FIGURE 1:**
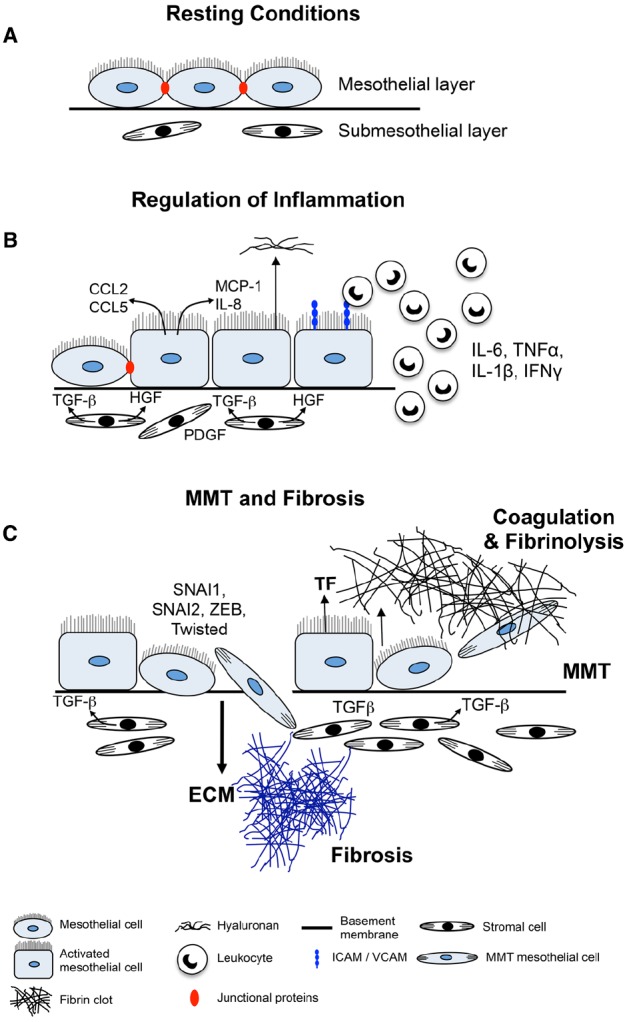
**Mechanisms of mesothelial cell-induced fibrosis. (A)** Normal serosa. Mesothelial cells rest on a basement membrane with submesothelial stromal cells embedded within ECM. **(B)** Inflamed serosa. Activated mesothelial cells secrete inflammatory mediators and growth factors into the serosal fluid and submesothelial compartment. Chemokines and other inflammatory mediators produced by the mesothelial cells attract inflammatory and immune cells to the site of injury and activate submesothelial stromal cells. Mediators produced by activated mesothelial cells and submesothelial stromal cells induce mesothelial cells to become more cuboidal, break cell–cell junctions, separate and expose underlying basement membrane and ECM. **(C)** MMT and fibrosis. Mesothelial cells secrete TF to induce coagulation and deposition of a fibrin matrix. Stromal and inflammatory cells secrete MMT-promoting factors that induce conversion of mesothelial cells into (myo)fibroblasts which migrate into the surrounding ECM and together with resident stromal cells form fibrotic foci.

This review will focus on aspects of the mesothelium that contribute to fibrosis including coagulation and fibrinolysis, inflammation, ECM production, and EMT/MMT, and discuss some common fibrotic conditions attributed to changes in mesothelial cell structure and function (Figure 1).

## Mesothelial Cell Functions

### Coagulation and Fibrinolysis

Mesothelial cells are important regulators of fibrin levels in the serosal cavities following injury (Rougier et al., 1998; Mutsaers et al., 2004). Fibrin deposition is an early step in normal wound repair but persistence of fibrin can lead to fibrosis and post-operative adhesion formation. For example in serosal cavities, the denudation of the mesothelium can cause impairment in the regulation of fibrinolytic activity by mesothelial cells and an accumulation of fibrin. If this fibrin is not removed it is replaced by granulation tissue that will be substituted by dense fibrous tissue (Dobbie and Jasani, 1997; Yung and Chan, 2012).

The regulation of fibrin deposition by mesothelial cells is mediated by the secretion of both procoagulant and fibrinolytic enzymes. Procoagulant activity is due to production and regulation of tissue factor (TF), the main cellular initiator of the extrinsic coagulation cascade. TF is produced by mesothelial cells (Bottles et al., 1997; Dobbie and Jasani, 1997) and complexes with other coagulation cascade proteins to activate thrombin which in turn cleaves serum fibrinogen to form fibrin. This is regulated by TF pathway inhibitor (TFPI), also produced by mesothelial cells (Bajaj et al., 2000). It has been shown in pleural injury that a relative excess of TF activity is expressed so that the inhibitory capacity of TFPI and other endogenous inhibitors are exceeded and local coagulation is thereby promoted (Bajaj et al., 2000).

Fibrinolytic activity is mediated through secretion of tissue plasminogen activator (tPA), urokinase PA (uPA), and uPA receptor (uPAR), and their inhibitors plasminogen activator inhibitors (PAI)-1 and PAI-2. The clearance of fibrin is based on the balance of the expression of the components of the fibrinolytic system and their net influence on local fibrinolytic activity (Mutsaers et al., 2004).

Mesothelial cells express tPA, uPA, uPAR, and PAI-1(Idell et al., 1992; Ivarsson et al., 1998). In the pleura, all these components, together with plasminogen, the substrate for uPA and tPA, can be detected in the pleural fluid (Idell et al., 1991). The fibrinolytic pathway can be activated directly by tPA or via expression of uPAR on the surface of pleural mesothelial cells (Shetty et al., 1995a), lung fibroblasts (Shetty and Idell, 1998), and macrophages (Sitrin et al., 1996). Because uPA binds to uPAR with high affinity, the bound form retains PA activity even in the presence of protease inhibitors (Higazi et al., 1998). Apart from its fibrinolytic properties, uPA can also initiate signaling through uPAR which also contributes to the pathogenesis of serosal inflammation and repair. uPA can upregulate uPAR in mesothelial cells and also contributes to chemotactic and mitogenic responses induced by pleural mesothelial cells and lung fibroblasts (Shetty et al., 1995a,b).

Both pro- and anti-fibrinolytic mediators are regulated by inflammatory factors including lipopolysaccharide, tumor necrosis factor alpha (TNF-α), and interleukin (IL)-1 and fibrogenic mediators such as transforming growth factor beta (TGF-β) and thrombin (Tietze et al., 1998). If the fibrinolytic capacity is insufficient and fibrin accumulation is not resolved, fibrous adhesions/plaques form between opposing serosal surfaces (Sulaiman et al., 2002).

Control of fibrin deposition and lysis is particularly important in the pleura. Cytokines implicated in the pathogenesis of pleural injury, including TNF-α, can upregulate uPAR expression at the surface of cell types involved in pleural injury (Yoshida et al., 1996) and thereby influence local remodeling of transitional fibrin. Exposure of mesothelial cells to asbestos can also influence uPAR expression (Perkins et al., 1999). The fibrinolytic system can also be controlled transcriptionally and post-transcriptionally through changes in uPAR mRNA stability and translational control. The regulatory mechanism involves the interaction and destabilization of uPAR mRNA through formation of *cis–trans* complexes between uPAR mRNA binding proteins and specific sequences of uPAR mRNA (Shetty et al., 2008).

### Mesothelial Cells Regulate Inflammation

Mesothelial cells play a critical role in the modulation of serosal inflammation through their ability to synthesize cytokines/chemokines, growth factors, ECM proteins, and intracellular adhesion molecules as well as their ability to present antigen. When the serosa is challenged by infection or agents such as dialysis fluid or asbestos, there is a massive influx of leukocytes from the vasculature into the serosal space (Jantz and Antony, 2008; Yung and Chan, 2012). Mediators released from activated macrophages such as TNF-α, IL-1β, and interferon gamma (IFN-γ) stimulate mesothelial cells to produce cytokines such as monocyte chemotactic protein-1 (MCP-1) also known a chemokine (C–C motif) ligand 2 (CCL2), RANTES also known as CCL5 and IL-8 also known as chemokine (C–X–C motif) ligand 8 (CXCL8) and adhesion molecules such as intercellular adhesion molecule-1 (ICAM-1), vascular cellular adhesion molecule-1 (VCAM-1), E-cadherin, N-cadherin, CD49a, CD49b, and CD29 (Jonjic et al., 1992; Cannistra et al., 1994; Liberek et al., 1996; van Grevenstein et al., 2007) to further recruit more leukocytes to the site of injury and facilitate leukocyte adherence and migration across the mesothelium (Liberek et al., 1996; Jantz and Antony, 2008; Yung and Chan, 2009, 2012).

Mesothelial cells also mediate inflammation through the local synthesis of hyaluronan (Yung and Chan, 2009, 2012), which is able to sequester free radicals and initiate tissue repair responses (Yung et al., 1994, 1996, 2000; Yung and Chan, 2007). Synthesis of hyaluronan fragments are increased by exposure to IL-1β, IL - 6, TNF-α, TGF-β_1_, and platelet-derived growth factor (PDGF; Yung et al., 1996) and can activate the inflammatory cascade in mesothelial cells by inducing IL-8 and MCP-1 production via activation of the NF-κB signaling pathway (Haslinger et al., 2001). In the peritoneum, induction of these inflammatory cytokines by long-term exposure to peritoneal dialysis (PD) fluid may promote the development of chronic peritoneal inflammation, leading to long-term peritoneal damage and exacerbation of the fibrotic pathway.

Mesothelial cells also contribute to controlling inflammation both in normal and inflamed tissue by producing cyclooxygenase (Baer and Green, 1993) and metabolizing arachidonic acid to release prostaglandins and prostacyclin (Stylianou et al., 1990; Topley et al., 1994).

### Mesothelial Cells Produce Extracellular Matrix

Mesothelial cells secrete a variety of ECM molecules, which physiologically are important for cell function and repair of serosal membranes. Mesothelial cells synthesize ECM molecules including collagen types I, III, and IV, elastin, fibronectin, laminin, and proteoglycans (Rennard et al., 1984; Laurent et al., 1988; Owens and Grimes, 1993; Milligan et al., 1995; Yung et al., 1995; Xiao et al., 2010) and they can also regulate ECM turnover by secreting matrix metalloproteinases and tissue inhibitors of metalloproteinases (Ma et al., 1999). In culture, mesothelial cells can be further stimulated to produce ECM when exposed to peritoneal effluent from patients with acute peritonitis (Perfumo et al., 1996) or various cytokines and growth factors such as IL-1β, TNF-α, epidermal growth factor (EGF), PDGF, and TGF-β (Owens and Grimes, 1993; Owens and Milligan, 1994; Zhang et al., 2005).

The renin–angiotensin system also stimulates ECM production (Noh et al., 2005). During PD and peritonitis, angiotensin II levels are increased. This promotes mesothelial cell production of fibronectin via the induction of the ERK1/2 and MAPK pathways thereby contributing to peritoneal injury and inflammation (Kiribayashi et al., 2005). The increased production of fibronectin by mesothelial cells can also be induced by the presence of advanced glycation end products (AGEs; Tong et al., 2012).

### Epithelial-to-Mesenchymal Transition

Mesothelial cells undergo MMT, a similar process to EMT in epithelial cells (López-Cabrera, 2014). EMT is a well characterized process, involving a number of overlapping and sequential events that require the appropriate spatiotemporal expression, interaction, and modification of a number of intra- and extra-cellular factors to cause a change in cell phenotype (Thiery et al., 2009). The process is controlled primarily by three main families of transcription factors: zinc finger Snail (SNAI1, SNAI2) basic helix–loop–helix (Twisted1), and ZEB (ZEB1, ZEB2; Thiery et al., 2009). Epithelial cells initially lose cell–cell junctions by down-regulating E-cadherin and other junctional proteins, reduce attachment to the basal lamina and subsequently lose apical–basal cell polarity. With cell migration and invasion of the basement membrane and a change in cytoskeletal components, a full change to a mesenchymal phenotype occurs. Expression of a multitude of mesenchymal markers, including alpha smooth muscle actin (α - SMA), EDA-fibronectin, vimentin, and fibroblast specific protein - 1 (FSP-1), is proposed as an unequivocal indicator of EMT (Zeisberg and Neilson, 2009). The fibrogenic mediator TGFβ is the most well described inducer of EMT whereas bone morphogenic protein-7 has been identified as a repressor in certain tissues (Zeisberg and Kalluri, 2004). MicroRNAs have emerged as important regulators of EMT as they are able to target multiple signaling pathways (Lamouille et al., 2013).

#### Evidence of MMT

Mesothelium-specific genetic lineage tracing studies in mice have clearly demonstrated that during development, mesothelial cells contribute to smooth muscle in the developing vasculature of the gut, heart, liver, and lungs through EMT (Wilm et al., 2005; Cai et al., 2008; Que et al., 2008; Zhou et al., 2008, 2010; Asahina et al., 2011), which will subsequently be referred to as MMT. The transcription factor Wilms tumor-1 (WT-1), expressed by mesothelium regulates its functional properties during development. During lung development, WT-1 expressing mesothelial cells migrate into the lung parenchyma and undergo a transition to form subpopulations of bronchial smooth muscle cells, vascular smooth muscle cells, and fibroblasts (Que et al., 2008; Dixit et al., 2013), through the action of sonic hedgehog signaling (Dixit et al., 2013). This process has also been shown to occur in the adult (Wada et al., 2003; Kawaguchi et al., 2007; van Tuyn et al., 2007). For example, Lachaud and colleagues (Lachaud et al., 2013) isolated murine uterine-derived mesothelial cells and stimulated them to undergo MMT and become functional vascular smooth muscle-like cells expressing smoothelin-B typical of contractile cells.

*In vitro*, numerous groups have shown upregulation of mesenchymal markers and downregulation of junctional components by human mesothelial cells following exposure to various injurious agents. TGF-β1 induced MMT in human mesothelial cell cultures isolated from the pleura, omentum, or mesenteric tissue, with evidence of downregulation of junction components (E-cadherin, ZO-1), upregulation of mesenchymal markers (α-SMA), and deposition of ECM (Yang et al., 2003; Nasreen et al., 2009). A number of studies have shown an upregulation of transcription factors in mesothelial cells associated with MMT (SNAI1/SNAI2, ZEB1/2, Twist1) following exposure to TGF-β1 as well as other cytokines including hepatocyte growth factor (HGF), PDGF, and IL-Iβ (Liu et al., 2008; Strippoli et al., 2008; Patel et al., 2010b; Zhou et al., 2013). Lipopolysaccharide, a derivative of the bacterial cell wall, has also been found to induce MMT and is proposed to be a mechanism whereby peritonitis is linked to peritoneal fibrosis (Liu et al., 2014b).

#### MMT and Fibrosis

*In vivo*, a number of studies have reported the importance of mesothelial cells in the development of fibrosis following injury. In a rat peritoneal scrape injury model, DiI-labeled rat mesothelial cells injected into the peritoneal cavity were found to incorporate into the mesothelial layer, eventually appearing in the subserosa (Foley-Comer et al., 2002). Furthermore, adenovirus-mediated overexpression of TGF-β1 in the lung and peritoneum induced fibrosis in mice that was associated with MMT; reduced E-cadherin and increased COL1, α-SMA, MMP-2, and 9 (Margetts et al., 2005; Decologne et al., 2007). These changes are likely to be mediated by both Smad3-dependent and independent signaling pathways (Patel et al., 2010a). Such findings confirm the ability of mesothelial cells to undergo MMT following damage. The possibility that there may be a genetic basis to this process was demonstrated by a study investigating mouse strain differences in susceptibility to TGF-β1-induced peritoneal fibrosis. Interestingly, an increase in markers of MMT was associated with enhanced peritoneal fibrosis in the susceptible mouse strain (C57/Bl6) whereas the resistant strain (SJL) showed minimal response (Margetts et al., 2013).

Of note, it is apparent that MMT may not just be of relevance to peritoneal fibrosis and that a similar process occurs in other organs/tissues and possibly re-activating developmental programs in the adult. For instance, Li et al. (2013), using conditional cell lineage murine studies, demonstrated that hepatic stellate cells and myofibroblasts are derived from mesothelial cells expressing WT-1 during liver fibrogenesis. In addition, a study using similar techniques in mice found that WT-1 positive pleural mesothelial cells migrated into the lung parenchyma leading to lung fibrosis following TGF-β1 treatment (Karki et al., 2014). Lansley and colleagues (Lansley et al., 2011) also demonstrated that mesothelial cells undergo MMT during differentiation into osteoblast-like and adipocyte-like cells in culture, and suggested mesothelial cells may have progenitor/stem cell-like properties.

## The Mesothelial Cell in Fibrotic Disorders

### Pleural Fibrosis

Pleural fibrosis resembles fibrosis in other tissues and may be the consequence of an organized hemorrhagic effusion, tuberculous effusion, empyema, asbestos-related pleuracy and chronic inflammatory conditions such as systemic lupus erythematosus, rheumatoid arthritis, and scleroderma (Idell, 2008; Schneider et al., 2012). In addition, certain medications have also been associated with the development of pleural fibrosis including procainamide, hydralazine, isoniazid (Huggins and Sahn, 2004), and targeted therapies such as tyrosine kinase inhibitors imatinib and dasatinib (Barber and Ganti, 2011). Pleural fibrosis can manifest itself as discrete localized lesions (pleural plaques) or diffuse pleural thickening and fibrosis. The mesothelial cell plays an important role in the fibrotic process through interaction with inflammatory cells, profibrotic mediators and both the coagulation and fibrinolytic pathways.

Fibrin is not normally present in the pleural space but rapidly accumulates in response to pleural injury. This was shown in an experimental rabbit model using intrapleural administration of tetracycline (TCN) to induce an acute pleural injury. Fibrin coated the pleural surfaces soon after injury and induced a peripheral pneumonitis with an exudative pleural effusion, leading to the formation of fibrinous adhesions within the exudative effusion (Strange et al., 1995; Idell et al., 1998, 2002). These fibrinous adhesions were rapidly remodeled with deposition of collagen within a few days (Miller et al., 1999). This model parallels the temporal course of loculation and fibrosis often observed in patients with complicated parapneumonic effusions (Light, 2003).

Fibrinolytic therapy, predominantly with streptokinase and urokinase (Bergh et al., 1977; Bouros et al., 1997; Chin and Lim, 1997), is often used for pleural loculations associated with parapneumonic effusions or hemothoraces (Colice et al., 2000). The rapid appearance of intrapleural fibrin resembles fibrin deposition within the lung which can lead to accelerated pulmonary fibrosis, for example in severe cases of acute respiratory distress syndrome (ARDS; Idell, 1995). TF is released locally by mesothelial cells and other resident and inflammatory cells into the pleural space (Drake et al., 1989; Idell et al., 2001) together with various coagulation factors including TFPI.

Although the primary target cell for pleural fibrosis is thought to be the subpleural fibroblast, studies have shown the importance of mesothelial cells in the pleural fibrotic response. A number of agents can induce fibrosis, including infection, radiation, and inorganic particles such as talc and asbestos (Dail and Hammar, 1994; Rom, 1998a,b). It is unclear how asbestos fibers induce subpleural fibroblasts and mesothelial cells to synthesize collagen but it is likely to be through the generation of cytokines, growth factors, and reactive oxygen species (ROS). ROS are cytotoxic and can stimulate fibroblasts to synthesize ECM components (Kamp and Weitzman, 1999) as well as induce expression of genes for profibrotic mediators such as TGF-β and TNF-α (Massague, 1996).

TGF-β is considered the most potent pro-fibrotic cytokine with a central role in the pathogenesis of many fibrotic diseases including pleural fibrosis. TGF-β stimulates collagen synthesis by mesothelial cells (Lee et al., 2003b), is present within pleural fluids in fibrosing forms of pleural injury (Lee and Lane, 2001) and induces pleural fibrosis when administered intrapleurally (Lee et al., 2000, 2003b). In addition, TGF-β lowers the ratio of matrix-degrading metalloproteinase-1 (MMP-1) to tissue inhibitors of metalloproteinases (TIMPs), promoting ECM accumulation (Ma et al., 1999). TGF-β has also been implicated in talc-induced pleurodesis, the most commonly used agent to induce pleurodesis (Lee et al., 2003a). Patients with tuberculous pleurisy also have elevated pleural fluid levels of TGF-β which was shown to correlate with increased levels of pleural thickening, an index of pleural fibrosis (Seiscento et al., 2007).

### Peritoneal Fibrosis Caused by Peritoneal Dialysis

Peritoneal dialysis (PD) is an effective renal replacement therapy used for patients with end stage kidney disease. The major disadvantage associated with this therapy is that PD solutions are bio-incompatible and contribute to the development of peritoneal fibrosis in most patients within two years of PD commencing (Garosi and Di Paolo, 2000, 2001; Yung and Chan, 2012). During PD, the mesothelial cells that line the peritoneum are exposed to a hypertonic environment with high glucose levels. As a consequence, mesothelial cells undergo structural and functional alterations that contribute to the development of fibrotic lesions in the peritoneum (Topley, 1998; Witowski et al., 2001; Lai and Leung, 2010; Yung and Chan, 2012).

Peritoneal biopsies taken from PD patients show a reactive mesothelium with enlarged, weakly adhesive, degenerated mesothelial cells with a reduced number of microvilli and alterations in the number of endoplasmic reticulum and micropinocytotic vesicles (Williams et al., 2002; Yung and Chan, 2012). In many patients, there is denudation of the mesothelial layer which is associated with vasculopathy and submesothelial thickening (Devuyst et al., 2002; Williams et al., 2003; Yung and Chan, 2009; Tomino, 2012). PD patients with subsequent peritonitis show even more pronounced mesothelial degeneration and a more prominent exfoliation of mesothelial cells (Verger et al., 1983; Di Paolo et al., 1986; Yung and Chan, 2012). In these patients, there is also an acute infiltration of inflammatory cells into the submesothelium that contribute to the thickening of this layer (Margetts et al., 2002b; McLoughlin et al., 2004; Dioszeghy et al., 2008).

Alterations to the structure of the peritoneum may be attributed to changes in mesothelial cell proteoglycan production (Yung et al., 2004; Osada et al., 2009; Tomino, 2012). Proteoglycans are anionic macromolecules and important components of ECM in the peritoneum (Iozzo, 2005). Mesothelial cells produce a number of small proteoglycans including perlecan, biglycan, and decorin (Yung et al., 1995, 2007; Yung and Chan, 2009). As PD progresses, there is an induction of versican while decorin and perlecan levels are reduced. These changes are associated with peritoneal ECM remodeling and expansion of the submesothelium (Yung et al., 2004; Osada et al., 2009). However, direct evidence for a role of these proteoglycans in serosal remodeling has yet to be demonstrated.

The chronic exposure of peritoneal mesothelial cells to high levels of glucose and glucose degradation products contributes to loss of the mesothelial layer by decreasing mesothelial cell viability (Witowski et al., 2001) and altering normal mesothelial cell function through the induction of proinflammatory factors such as vascular endothelial growth factor (VEGF) and TGF-β1 (Ciszewicz et al., 2007; Baroni et al., 2012). VEGF is associated with neoangeogenesis (Combet et al., 2000; Szeto et al., 2004; Yung and Chan, 2012) and the down-regulation of the mesothelial cell intercellular tight junction proteins ZO-1, occludin, and claudin-1 (Lai and Leung, 2010) while TGF-β1 is associated with lymphangiogenesis (Kinashi et al., 2013), the promotion of MMT (Margetts et al., 2005; López-Cabrera, 2014), and the production of collagen type I, III (Kim et al., 2008), and IV (Mateijsen et al., 1999).

The fibroblast-like characteristics induced in mesothelial cells that have undergone MMT allow these cells to invade into the submesothelial stroma where they contribute to angiogenesis, fibrosis, and ultrafiltration failure (Lai and Leung, 2010). These cells are often observed in patients who have undergone PD for more than 12 months (Yanez-Mo et al., 2003). MMT is associated with polymerization of the actin cytoskeleton and an increase in hyaluronan (Yung et al., 2000; Yung and Chan, 2007, 2009) and is mediated by proinflammatory factors such as IL-1β, EGF, HGF (Yung and Chan, 2009), AGEs and their receptor RAGE (De Vriese et al., 2006). The prolonged expression of these factors during peritoneal inflammation delays the regression of mesothelial cells back to their epithelial phenotype thereby promoting fibrotic changes in the peritoneum. Other factors recently identified to be associated with MMT include MCP-1 (Lee et al., 2012), ROS (Liu et al., 2012), and the small non-coding regulatory microRNAs miR-589 (Zhang et al., 2012), miR-30a (Zhou et al., 2013), miR-30b (Liu et al., 2014a), and miR-200c (Zhang et al., 2013).

Recently, JAK/STAT signaling was also identified as a mediator of PD-induced peritoneal membrane changes (Dai et al., 2014). Twice daily PD fluid infusions in rats for 10 days induced phospho-JAK, mesothelial cell hyperplasia, inflammation, fibrosis, and hypervascularity. These changes were attenuated following the administration of a JAK1/2 inhibitor. These findings are consistent with recent observations in a mouse model of lung fibrosis where blocking STAT3 attenuated the fibrotic response (O’Donoghue et al., 2012). Therefore, targeting the JAK/STAT signaling pathway may be a novel therapeutic strategy used to reduce PD related peritoneal changes that contribute to the development of peritoneal fibrosis in patients.

The processes by which the peritoneum repairs following PD associated injury are yet to be fully defined. Viable mesothelial cells are exfoliated into the peritoneal cavity during PD and it is likely that these cells re-populate and restore the damaged mesothelium (Yung and Chan, 2009; Tomino, 2012; Yung and Chan, 2012). Therefore it has been proposed that mesothelial cell transplantation could be used therapeutically to regenerate the PD injured mesothelium. Studies have shown that mesothelial cell transplantation is feasible in animals and humans (Di Paolo et al., 1991; Hekking et al., 2003) and that genetically modified mesothelial cells can also be used to deliver proteins critical to the healing process (Nagy et al., 1995). However, in one study the transplantation of mesothelial cells in rats was shown to activate the peritoneum and induce inflammation (Hekking et al., 2005) and recently, the morphology of the mesothelial cell was shown to be important for cell therapy used for peritoneal regeneration (Kitamura et al., 2014). Mesothelial cells harvested from the PD effluent of patients were separated based on morphology into epithelial-like and fibroblastic-like cells and transplanted into nude mice with an injured peritoneum. The mice transplanted with epithelial-like cells showed very few adhesions and exhibited no thickening of the peritoneum. However, transplantation of fibroblast-like cells did not inhibit peritoneal adhesion or thickening, highlighting the need for further optimization before this approach can be trialed in patients. Other cell sources that may be used for mesothelial repair include bone marrow derived cells (Sekiguchi et al., 2012), adipose-derived stem cells (Kim et al., 2014), and mesenchymal stem cells (Wang et al., 2012; Ueno et al., 2013). Alternative therapeutic strategies being investigated to reduce mesothelial cell-mediated inflammation and prevent peritoneal fibrosis include targeting TGFβ1-mediated mechanisms (Hung et al., 2001, 2003; Yung et al., 2001; Margetts et al., 2002a; Fang et al., 2006; Tomino, 2012; Jang et al., 2013), reducing mesothelial cell production of fibronectin (Tong et al., 2012; Zhang et al., 2014) developing a more bio compatible PD solution (Bajo et al., 2000; Le Poole et al., 2005), altering PD daily dwelling time (Lee et al., 2014), and stimulating fibrinolytic agents (Haslinger et al., 2003).

### Postoperative Adhesions

The formation of postoperative intra-abdominal and pelvic adhesions is a significant clinical and surgical problem. Adhesions are bands of fibrous tissue that form between apposing tissue and organs usually arising as a result of injury sustained during surgery (Dizerega and Campeau, 2001). They are a leading cause of chronic pelvic pain, intestinal obstruction, and female infertility (Rajab et al., 2009). The most severe consequence of adhesion formation is small bowel obstruction which can occur up to 20 years or more after the initial surgical procedure (Isaksson et al., 2014) and is associated with mortality rates ranging between 3% and 30% (Ellis, 1997). Postoperative adhesions have been reported to occur in up to 93% of patients undergoing abdominal surgery (Ellis, 1997). A substantially increased risk of post-surgical complications is also likely where adhesions are present as a result of previous surgery (Trochsler and Maddern, 2014).

Adhesions are thought to occur when there is dysregulation of the normal serosal healing process (Dizerega and Campeau, 2001). Many cell types including macrophages, lymphocytes, granulocytes, and fibroblasts play important roles in serosal repair (Brochhausen et al., 2012a), however the mesothelial cell is central to this process but may also play a critical role in the development of adhesions following injury (Attard and MacLean, 2007). As discussed, mesothelial cells secrete a variety of coagulation and inflammatory mediators following serosal injury (Brochhausen et al., 2012b) and it is these factors that are the essential inducers of adhesion development.

Following serosal trauma (such as during surgery), the mesothelial layer is disrupted resulting in brief vasoconstriction followed by increased vascular permeability and chemotaxis of inflammatory cells to the site of injury (Alonso Jde et al., 2014). Mesothelial cells stimulate fibrin deposition through the production of TF and themselves become embedded in the developing fibrin scaffold (Boland and Weigel, 2006). Under normal conditions the fibrin is degraded following release of fibrinolytic mediators from the mesothelial cells, such as tPA, but if there is a persistent fibrinolytic imbalance, there is subsequent deposition of ECM components by mesothelial cells, fibroblasts, and myofibroblasts. Ultimately this results in the formation of fibrin bands between tissues and organs which then become organized into fibrous adhesions (Alonso Jde et al., 2014).

Detrimental effects of surgical techniques on peritoneal mesothelial cells have been reported which are thought to contribute to adhesion formation (Brochhausen et al., 2012a). For example, use of the common insufflation agent carbon dioxide gas (CO_2_) as well as the amount of insufflation pressure used during laparoscopy can result in morphological and biochemical changes to mesothelial cells and can cause hypoxia and dehydration (Molinas and Koninckx, 2000; Ott, 2001). Therefore, several changes have been made to surgical techniques in order to prevent the mesothelial cell denudation and bleeding that also form the basis of peritoneal adhesion formation. These have included development of new microsurgical techniques (minimally invasive surgery), the use of specialized equipment and unpowdered gloves (Brochhausen et al., 2012a) and humidifying and changing the temperature and composition of the gases used for laparoscopy (Schlotterbeck et al., 2011; Binda et al., 2014).

Currently, there are no definitive strategies to prevent the formation of adhesions during surgery. Many methods have been developed and tested using a variety of post-surgical adhesion animal models (Verco et al., 2000; Gorvy et al., 2005; Lee et al., 2005; Oh et al., 2005; Kement et al., 2011) as well as in human clinical trials (Pados et al., 2010) but with varying degrees of success. Addition of surgical barriers that provide anti-adhesive separation of denuded serosal tissues have proved beneficial but none completely prevent adhesion development in all patients (Alonso Jde et al., 2014).

Strategies targeting the pathophysiological mechanisms involved in dysregulated serosal repair, such as the coagulation and inflammatory pathways, have also been trialed in an effort to prevent adhesion formation. Many anti-inflammatory and anti-coagulant substances have been used both systemically and locally including steroids (Avsar et al., 2001), cyclo-oxygenase inhibitors (Lee et al., 2005; Oh et al., 2005), heparin (Kutlay et al., 2004; Kement et al., 2011), and tPA (Dorr et al., 1990; Irkorucu et al., 2009) but to date, none of these agents have shown significant promise (Brochhausen et al., 2012a).

Studies have also examined the effect of mesothelial cell transplantation on preventing adhesion formation and this approach has shown some promise (Bertram et al., 1999; Takazawa et al., 2005; Asano et al., 2006; Kawanishi et al., 2013). However, how this approach can be used routinely in patients still needs to be determined. Clearly a better understanding of the mechanisms underlying adhesion formation is therefore critical to developing novel approaches to prevent their formation.

### Idiopathic Pulmonary Fibrosis

Interstitial lung diseases (ILDs) represent a collection of heterogeneous parenchymal lung disorders characterized by inflammation and fibrosis that lead to impairment of gas-exchange in the lungs. Approximately 50% of ILDs have unknown etiology, of which idiopathic pulmonary fibrosis (IPF) is a well-defined subset.

Histologically, the lungs in IPF demonstrate a pattern of usual interstitial pneumonia, which includes septal thickening, honeycombing, fibroblastic foci, and minimal interstitial inflammation (Raghu et al., 2011). IPF occurs predominantly from middle age onwards affecting five million people worldwide (Meltzer and Noble, 2008). It is a debilitating and ultimately lethal disease, with a mortality rate worse than that seen with many cancers (Nicholson et al., 2000). It has a median survival of only 2–3 years from diagnosis (Raghu et al., 2011), and there is currently no known cure. Recent phase III trial results showed that current drugs such as pirfenidone and nintedanib could only slow the progression of the disease (King et al., 2014; Richeldi et al., 2014). Pirfenidone works through downregulation of growth factor and procollagen I and II production and nintedanib is a small molecule tyrosine kinase inhibitor that blocks receptors for VEGF, PDGF, and FGF.

The pathogenesis of IPF remains poorly understood although the mechanisms driving the fibrotic response are often considered to follow a similar pathway to other forms of tissue fibrosis where there is a chronic progression of the repair response resulting in excessive deposition of ECM without resolution (Thannickal et al., 2004).

In IPF, the myofibroblast, characterized by α-SMA and vimentin expression, is recognized as the effector cell contributing to the deposition of ECM (Kuhn and McDonald, 1991), mainly types I and III collagen (Madri and Furthmayr, 1980). However, the cellular origin of the lung myofibroblast remains controversial and a combination of different cell types likely serves as precursors of myofibroblasts. A number of cellular sources of myofibroblast have been proposed, including existing peribronchial and perivascular adventitial fibroblasts, alveolar epithelial cells, bone marrow-derived cells, tissue-resident cells, and pericytes (Phan, 2002; Hinz et al., 2007; Greenhalgh et al., 2013).

As previously discussed, mesothelial cells can be induced to undergo MMT and transition into myofibroblasts. Decologne and colleagues (Decologne et al., 2007) used adenoviral gene transfer of TGF-β to the pleural mesothelium in rats and showed that as well as development of a progressive pleural fibrosis, the pleural fibrosis extended into the lung parenchyma supporting a possible role for mesothelial cells in pulmonary fibrosis. More recent mouse models of fibrogenic lung injury have also supported this observation by showing that mesothelial cells invade the lung parenchyma and adopt a myofibroblast phenotype after intratracheal TGF-β1 administration, leading to fibrosis (Zolak et al., 2013; Karki et al., 2014). This was recently shown to be mediated through the TGF-β1-Smad2/3 signaling pathway (Chen et al., 2015). Blocking this pathway using novel TGF - β regulators, such as the nuclear receptor NR4A1, are likely to block MMT and attenuate tissue fibrosis (Palumbo-Zerr et al., 2015).

As a further validation of the *in vitro* and *in vivo* findings, immunohistochemical analysis of human IPF lung sections showed Wilms tumor-1 (WT-1)-positive mesothelial cells in the pleura and lung parenchyma, which corresponded with immunostaining of the mesothelial cell marker calretinin (Zolak et al., 2013; Karki et al., 2014). In contrast, lung tissue sections from patients with chronic obstructive pulmonary disease, cystic fibrosis, and pulmonary arterial hypertension were all negative for WT-1. Collectively, these studies indicate potential contributions of pleural mesothelial cells as a source of myofibroblast in IPF and possibly a new avenue to identify therapeutic targets.

## Conclusion

Mesothelial cells clearly play an important role in serosal homeostasis and repair following injury, but following a breakdown in the normal regulatory mechanisms, mesothelial cells can also contribute to the development of tissue fibrosis. The mechanisms underlying this process are slowly being elucidated but more research is needed to investigate how mesothelial cells interact with their local environment and to identify ways to limit fibrosis and promote normal repair.

### Conflict of Interest Statement

The authors declare that the research was conducted in the absence of any commercial or financial relationships that could be construed as a potential conflict of interest.
